# Remnant cholesterol and suicide attempts in untreated first-episode major depressive disorder

**DOI:** 10.3389/fpsyt.2025.1493509

**Published:** 2025-03-04

**Authors:** Ping Xu, Cheng Fan, Mingxing Yan, Junjun Liu, Xiangyang Zhang

**Affiliations:** ^1^ Department of Psychiatry, Nanjing Lishui District Psychiatric Hospital, Lishui, China; ^2^ Nanjing Department of Psychiatry, The Third People’s Hospital of Lishui District, Lishui, China; ^3^ Department of Psychiatry, Nanjing Meishan Hospital, Nanjing, China; ^4^ Medical College of Soochow University, Suzhou, China; ^5^ Suzhou Guangji Hospital, The Affiliated Guangji Hospital of Soochow University, Suzhou, China; ^6^ Chinese Academy of Sciences (CAS) Key Laboratory of Mental Health, Institute of Psychology, Chinese Academy of Sciences, Beijing, China

**Keywords:** remnant cholesterol, major depressive disorder, suicide attempts, non-linear relationship, untreated first-episode

## Abstract

**Objective:**

The objective of this research was to investigate the relationship between remnant cholesterol (RC) levels and suicide attempts (SA) made by Chinese patients with untreated first-episode major depressive disorder (UFE MDD).

**Methods:**

This study included 1718 patients with UFE MDD. Demographic, clinical characteristics, and blood lipid parameters were collected. The 17-item Hamilton Depression Rating Scale (HAMD), the 14-item Hamilton Anxiety Rating Scale (HAMA), and the positive subscale of the Positive and Negative Syndrome Scale (PANSS) were used to assess their depression, anxiety, and psychotic symptoms, respectively. Multivariable binary logistic regression analysis was used to estimate the association between RC and the risk of SA. A two-piecewise linear regression model was used to investigate the threshold effects if non-linear associations existed.

**Results:**

Univariate logistic regression analysis showed a significant positive correlation between RC and SA, but after controlling for confounding factors, the association between them was not statistically significant. After dividing the RC into quartiles, only the RC in the Q4 group was significantly positively correlated with suicide attempts (OR = 1.73, 95% CI: 1.13-2.65, P = 0.012, vs. Q1) in a fully adjusted model. Curve fitting analysis also showed a nonlinear relationship between RC and suicide attempts with an inflection point at 1.99 mmol/L in RC. On the left of the inflection point, a significant positive correlation was observed between RC and SA (OR: 1.36, 95% CI: 1.09-1.69, p=0.006). However, on the right of the inflection point, no significant correlation was found (OR: 0.79, 95% CI: 0.55-1.14, p=0.214).

**Conclusion:**

This study demonstrates a non-linear association between RC levels and SA in patients with untreated first-episode major depressive disorder. When RC was less than 1.99 mmol/L, they showed a significant positive correlation.

## 1 Background

According to the World Health Organization, over 800,000 individuals commit suicide annually, making it a serious global public health risk (1). Among those who die by suicide, more than 90% suffer from mental disorders, particularly depression, accounting for 59–87% of all suicide cases (2). Notably, the incidence of suicide attempts (SA) is 20 times that of completed suicides (3). In the world, the lifetime prevalence of suicidal ideation is roughly 9.2%, whereas the lifetime rate of suicide attempts is 2.7% (4). Suicide risk is greatly increased by depression. Research indicates that individuals with major depressive disorder (MDD) have a lifetime rate of suicide attempts ranging from 16 to 33.7% (5), which is around twenty times greater than the general population (6). Furthermore, compared to people without the illness, those with severe depression are 7.34 times more likely to have tried suicide in the previous year, according to meta-analyses (7). In China, 23.7% of depression sufferers had attempted suicide at least once in their lifetime (8). Suicide attempts in MDD patients are associated with multiple factors, including gender (9), age (10), duration of illness (11), blood glucose and lipid abnormalities (12).

Lipid abnormalities play a crucial role in cardiovascular disease (CVD) pathogenesis (13, 14). While low-density lipoprotein cholesterol (LDL-c) reduction remains central to CVD prevention guidelines (15), substantial cardiovascular risk persists even after achieving target LDL-c levels (16). Emerging evidence suggests that remnant cholesterol (RC) may be a key contributor to this persistent risk (17). RC represents the cholesterol content of triglyceride-rich lipoproteins (TGRLs), including very-low-density lipoproteins (VLDL) and intermediate-density lipoproteins (IDL) during fasting, and chylomicron remnants in non-fasting states (18). Clinically, RC can be calculated using the formula: RC = total cholesterol - LDL-c - HDL-c (17). RC particles, being larger and more cholesterol-enriched than LDL-c particles, are more susceptible to macrophage uptake, promoting foam cell formation and atherosclerotic plaque development (19, 20). Systematic reviews have established RC as a reliable biomarker for atherosclerosis and cardiovascular risk assessment (21). Elevated RC levels have been associated with adverse outcomes, including ischemic stroke, myocardial infarction, and all-cause mortality (22, 23). As an emerging lipid parameter, RC may provide novel insights into lipid abnormalities’ pathogenicity and specificity compared to single lipid levels, making it a promising target for investigating the relationship between cardiovascular diseases and depression.

The relationship between blood lipid parameters and major depressive disorder (MDD) has been extensively documented. Epidemiological data indicate that among individuals with MDD, the prevalence of hypertriglyceridemia and hypercholesterolemia reaches 52.3% and 44.7%, respectively (12). Intriguingly, recent investigations have revealed a relationship between RC and MDD. A large-scale population-based study conducted in the United States (n = 8,263) demonstrated that patients with MDD exhibited significantly elevated RC levels compared to healthy controls (26.13 vs. 23.05 mg/dL, P < 0.001) (24). However, the relationship between cholesterol and suicidal behavior remains controversial. While some studies report inverse associations between cholesterol levels and suicide attempts (25, 26), others show positive correlations (27, 28), and some find no significant relationship (29, 30). These inconsistencies may stem from methodological variations, demographic differences, and environmental factors. Notably, the potential role of RC in suicidal behavior remains largely unexplored, warranting further investigation.

The goal of this research is to assess how RC and SA correlate in Chinese communities with untreated first-episode MDD patients. To our knowledge, this may be the first study on the association between suicide attempts and remnant cholesterol. Identifying new biomarkers that can predict suicide attempts in depressed patients is crucial for early detection and intervention of suicide risk, which will help reduce the burden on families and society.

## 2 Methods

### 2.1 Participants

A cross-sectional design was used in this investigation. From September 2016 to December 2018, 1718 outpatient patients were selected from the First Hospital of Shanxi Medical University. The following are the inclusion criteria for the study: (1) Han ethnicity; (2) between the ages of 18 and 60; (3) two knowledgeable clinical psychiatrists diagnosed MDD using the DSM-IV; (4) no antidepressants or antipsychotic medications are prescribed at the onset of depression symptoms; (5) the illness’s duration cannot exceed 24 months; (6) the lowest score on the 17 Hamilton Depression Scale (HAMD-17) is 24 points; (7) no thyroid hormone treatment or specific medication was previously administered. The exclusion criteria are as follows: (1) having substantial physical illnesses, such as severe infections or organic brain diseases; (2) having any other major DSM-IV axis I hurdles based on SCID; (3) being pregnant or lactating; and (4) abusing alcohol or other drugs, excluding smoking.

The sample size was calculated using the formula n = Z²p(1-p)/d² (31), where Z represents the standard normal variate at a 95% confidence level (1.96), d is the desired margin of error (0.05), and p denotes the estimated population proportion with the characteristic of interest. Based on a recent Chinese study reporting a 20.14% suicide attempt rate among depressed patients (32), the minimum required sample size was calculated to be 288 patients. Our final sample size of 1,718 participants substantially exceeded this minimum requirement, ensuring adequate statistical power for our analyses.

This study was approved by the Institutional Review Board of the First Hospital, Shanxi Medical University (No. 2016-Y27). Before beginning the study, each participant gave their free and informed consent in writing.

### 2.2 Sociodemographic features and anthropometric information

Gather general information and sociodemographic details about each patient, such as age, gender, marital status, education level, age of onset, and length of sickness, using a self-designed, structured questionnaire. With the aid of calibration tools and standard operating procedures, ascertain the anthropometric measurements (weight and height), systolic blood pressure (SBP), and diastolic blood pressure (DBP). The body mass index, or BMI, is calculated in this way: the ratio of weight (kg) to height (kg/m2).

### 2.3 Blood samples

Every individual was given an overnight fast before having a blood sample taken between 6 and 9 a.m. On that specific day, before 11 a.m., all samples were delivered to the hospital testing lab for measurement. Hormone evaluation was carried out by measuring thyroid stimulating hormone (TSH), free triiodothyronine (FT3), free thyroxine (FT4), anti-thyroglobulin (TGAb), and thyroid peroxidase antibody (TPOAb) using an automated clinical analyzer (Abbott, Longford, Ireland) and chemiluminescence particle immunoassay (CMIA). Use an automated analyzer (Architect c8000 system) to measure fasting blood sugar and lipid levels, such as cholesterol (TC), triglycerides (TG), low-density lipoprotein cholesterol (LDL-c), and high-density lipoprotein cholesterol (HDL-c). The following formula was used to determine remnant cholesterol (RC): RC = TC - HDL-c - LDL-c (17).

### 2.4 Clinical interview assessment

Two experienced psychiatrists diagnosed patients using the Chinese version of SCID-I/P (DSM-IV-TR-based). assessing each participant’s depression symptoms in-depth using the Hamilton Depression Scale (33). This 17-item scale uses three subscales, with 8 items scoring between 0 and 2 and the remaining 9 items scoring between 0 and 4 on five subscales (asymptomatic to severe symptoms). In this study, patients with a score of 24 or above were classified as having severe depressive symptoms. This scale is frequently used in China, and previous studies have shown its strong reliability and effectiveness (34). The Chinese Hamilton Anxiety Scale (HAMA) is used to assess the intensity of anxiety symptoms (35). The scale consists of 14 items, with scores ranging from 0 to 4 for each item (asymptomatic to severe symptoms) and total scores ranging from 0 to 56. Apply the Positive and Negative Syndrome Scale (PANSS) positive symptom subscale to assess the severity of psychiatric symptoms (36). Seven items make up the PANSS subscale, and each item uses a seven-point Likert scale. By applying a cutoff score of 15 points, separate the group into two categories: psychiatric and non-psychiatric (37). Suicide attempts were defined as any potentially self-hurt actions taken by the participants themselves, with some degree of intent to terminate their lives (2). Suicide attempt history was assessed through a face-to-face interview with the question, “Have you ever attempted suicide in your lifetime?” derived from the WHO/EURO multicenter study (38). Patients with UFE MDD who responded “yes” to this inquiry were regarded as having attempted suicide. Then, we probed more into the frequency, mode, and precise dates of suicide attempts. A total of 346 patients with UFE MDD were documented to have engaged in SA during their initial depressive episode. Among the SA, one patient made four attempts, two patients made three attempts, 26 patients made two attempts, and 317 patients made one attempt. Two licensed psychiatrists with over five years of clinical experience received pre-study training on assessment tools. Repeated assessments were used to evaluate the consistency between the raters of the total scores of the HAMD, HAMA, and PANSS-P, and the results showed an observer correlation coefficient greater than 0.8. Moreover, they are ignorant of the patient’s clinical state.

### 2.5 Statistical analysis

Whereas frequency and proportion (%) are used to describe categorical data, mean standard deviation (SD) is utilized to characterize continuous variables. RC and SA’s association can be assessed by using a binary logistic regression model. Measure the strength of the link by computing and providing the odds ratio (OR) and 95% confidence interval (CI) of the unadjusted and adjusted models. Finding multicollinearity between independent variables can be aided by excluding variables from the final model that have a variance inflation factor (VIF) > 5.0. The covariate is deemed a possible confounding factor in the final model if it modifies the RC estimate of attempted suicide by more than 10% or if it shows a significant connection (P < 0.10) with attempted suicide in MDD patients (39). Age, gender, duration of illness, psychotic symptoms, comorbid anxiety, HAMA, HAMD, TGAb, TSH, TPOAb, FBG, BMI, SBP, and DBP are among the confounding variables included in the fully adjusted model. Furthermore, in order to convert RC from continuous data to categorical categories and ascertain the trend’s P-value, we will perform sensitivity analysis. We investigated the link between RC and SA using a smooth graph. If there is a nonlinear link found, the threshold effect is calculated using a two-stage linear regression model based on the Generalized Estimation Equation (GEE). Regression models are compared using the log-likelihood ratio test (LLR). For every experiment, the statistical software program SPSS 25.0, EmpowerStats (http://www.empowerstats.com, X&Y Solution, Inc, Boston, Massachusetts, USA) and the statistical software programs R 4.3.0 (http://www.r-project.org, The R Foundation) were used for all analyses. Statistically significance was defined as a two-tailed p-value < 0.05.

## 3 Results

### 3.1 Baseline characteristics

A total of 1,718 UFE MDD patients met the criteria for enrollment and were included in this study, with 588 males (34.2%) and 1130 females (65.8%). Among the total study population, the overall prevalence of suicide attempts (SA) was 20.1% (346/1,718). When stratified by gender, the prevalence was 19.05% (112/588) in males and 20.71% (234/1,130) in females. Statistical analysis revealed no significant gender-based differences in SA prevalence (p > 0.05).

Participants were stratified into quartiles based on RC levels, with 430 in the first quartile (Q1), 428 in the second quartile (Q2), 431 in the third quartile (Q3), and 429 in the fourth quartile (Q4) of RC. The participants’ average age was 34.9 ± 12.4 years, with significant differences observed across quartiles (p = 0.011). The average duration of illness was 6.3 ± 4.7 months, with Q4 showing the longest duration (7.1 ± 4.9 months, p = 0.001). Comparison of participant characteristics across RC quartiles revealed significant associations between RC levels and age, duration of illness, age of onset, education level, marital status, HAMD, HAMA, TSH, TGAb, TPOAb, FT3, FBG, TC, TG, HDL-c, LDL-c, SBP, DBP, BMI, comorbid anxiety, psychotic symptoms, and suicide attempts (all p < 0.05). The gender distribution was 57.8% male and 42.2% female, with quartiles not significantly different from one another (p = 0.184). The baseline characteristics of the subjects are shown in **Table 1**, which is categorized by RC quartiles.

**Table 1:** Baseline characteristics of participants.

Variables	Total	RC quartile(mmol/l)				*P-* value
		Q1 (< 0.46)	Q2 (0.47 - 0.87)	Q3 (0.88 - 1.54)	Q4 (1.55 - 5.23)	
N	1718	430	428	431	429	
Age (years)	34.9 ± 12.4	33.3 ± 12.2	35.3 ± 12.6	35.9 ± 12.3	35.0 ± 12.6	0.011
Duration of illness (month)	6.3 ± 4.7	5.9 ± 4.7	5.9 ± 4.5	6.4 ± 4.8	7.1 ± 4.9	0.001
Age at onset (years)	34.7 ± 12.3	33.0 ± 12.1	35.1 ± 12.5	35.7 ± 12.2	34.8 ± 12.4	0.010
HAMD	30.3 ± 2.9	29.1 ± 3.0	29.6 ± 2.7	30.7 ± 2.8	31.8 ± 2.6	< 0.001
HAMA	20.8 ± 3.5	20.1 ± 3.3	20.3 ± 3.2	21.0 ± 3.6	21.8 ± 3.6	< 0.001
TSH (uIU/ml)	5.1 ± 2.6	4.1 ± 2.3	4.3 ± 2.3	5.2 ± 2.4	6.7 ± 2.4	< 0.001
TGAb (IU/L)	90.0 ± 238.3	60.5 ± 150.0	79.9 ± 173.0	99.7 ± 299.9	120.0 ± 287.8	0.002
TPOAb (IU/L)	72.3 ± 163.8	51.5 ± 114.3	68.6 ± 159.2	77.1 ± 181.6	91.9 ± 187.7	0.003
FT3 (pmg/dL)	4.9 ± 0.7	4.9 ± 0.7	4.8 ± 0.7	4.9 ± 0.7	5.0 ± 0.7	0.001
FT4 (pmg/l)	16.7 ± 3.1	17.1 ± 3.1	16.3 ± 3.1	16.6 ± 3.0	16.9 ± 3.1	< 0.001
TG (mmol/l)	2.2 ± 1.0	2.0 ± 1.0	1.7 ± 0.6	2.3 ± 0.8	2.7 ± 1.2	< 0.001
TC (mmol/l)	5.2 ± 1.1	4.4 ± 0.8	4.9 ± 0.8	5.4 ± 0.8	6.3 ± 0.9	< 0.001
HDL-c (mmol/l)	1.2 ± 0.3	1.3 ± 0.2	1.3 ± 0.2	1.2 ± 0.3	1.1 ± 0.3	< 0.001
LDL-c (mmol/l)	3.0 ± 0.9	3.0 ± 0.9	3.0 ± 0.8	3.1 ± 0.8	2.9 ± 0.9	0.012
BMI (kg/m^2^)	24.4 ± 1.9	24.4 ± 2.0	24.1 ± 1.8	24.5 ± 1.8	24.6 ± 2.0	0.001
FBG (mmol/l)	5.4 ± 0.6	5.3 ± 0.6	5.3 ± 0.6	5.4 ± 0.7	5.6 ± 0.6	< 0.001
SBP (mm/Hg)	119.5 ± 10.9	116.0 ± 11.0	118.5 ± 11.0	120.7 ± 10.1	122.7 ± 10.4	< 0.001
DBP (mm/Hg)	76.0 ± 6.7	74.5 ± 6.7	75.3 ± 6.7	76.5 ± 6.5	77.5 ± 6.7	< 0.001
Gender						0.184
Males	588 (57.8%)	142 (33.0%)	164 (38.3%)	136 (31.6%)	146 (34.0%)	
Females	1130 (42.2%)	288 (67.0%)	264 (61.7%)	295 (68.4%)	283 (66.0%)	
Marital status						< 0.001
Single	502 (34.2%)	155 (36.0%)	112 (26.2%)	101 (23.4%)	134 (31.2%)	
Marriage	1216 (65.8%)	275 (64.0%)	316 (73.8%)	330 (76.6%)	295 (68.8%)	
Education						0.002
Junior	413(24.1%)	96 (22.3%)	92 (21.5%)	99 (23.0%)	126 (29.4%)	
Senior	760 (44.2%)	187 (43.5%)	195 (45.6%)	218 (50.6%)	160 (37.3%)	
College	449 (26.1%)	116 (27.0%)	120 (28.0%)	96 (22.3%)	117 (27.3%)	
Postgraduate	96 (5.6%)	31 (7.2%)	21 (4.9%)	18(4.1%)	26 (6.0%)	
Suicide attempt						< 0.001
Yes	346 (20.1%)	49 (11.4%)	70 (16.4%)	89 (20.6%)	138 (32.2%)	
No	1372 (79.9%)	381 (88.6%)	358 (83.6%)	342 (79.4%)	291 (67.8%)	
Comorbid anxiety						0.005
Yes	1380 (80.3%)	332 (77.2%)	334 (78.0%)	345 (80.0%)	369 (86.0%)	
No	338 (19.7%)	98 (22.8%)	94 (22.0%)	86 (20.0%)	60 (14.0%)	
Psychotic symptoms						< 0.001
Yes	171 (10.0%)	27 (6.3%)	32 (7.5%)	51 (11.8%)	61 (14.2%)	
No	1547 (90.0%)	403 (93.7%)	396 (92.5%)	380 (88.2%)	368 (85.8%)	

The variables are presented as n (%) or the mean ± SD; RC, remnant cholesterol; HAMD, 17-item Hamilton Rating Scale for Depression; HAMA, 14-item Hamilton Anxiety Rating Scale; BMI, body mass index; FBG, fasting blood glucose; TC, total cholesterol; TG, triglyceride; HDL-c, high-density lipoprotein cholesterol; LDL-c, low-density lipoprotein cholesterol; TSH, thyroid-stimulating hormone; FT3, free triiodothyronine; FT4, free thyroxine; TGAb, thyroglobulin antibody; TPOAb, thyroid peroxidase antibody; SBP, systolic blood pressure; DBP, diastolic blood pressure.

### 3.2 Relationship between RC and SA in different models

The association between RC and suicide attempts (SA) was analyzed using binary logistic regression models. We present three models in **Table 2**: unadjusted, Model I, and Model II. In both the unadjusted and Model I (adjusted for age and gender), results showed a significant association between RC and SA (P < 0.001). In Model II, we found no significant correlation between RC and SA after correcting for other factors (OR = 1.14, 95% CI: 0.99 to 1.32, P = 0.072). After changing RC from a continuous to a categorical variable, we performed a sensitivity analysis. There was no discernible correlation between RC and SA in the Q2 and Q3 groups in Model II, with Q1 serving as the reference group (P > 0.05). But in the Q4 group, a significant correlation was noted (OR = 1.73, 95% CI: 1.13 to 2.65, P = 0.012).

**Table 2:** Relationship between RC and SA in different models.

Variable	N	Unadjusted Model		Model I		Model II	
		OR (95%CI)	*P-*value	OR (95%CI)	*P-*value	OR (95%CI)	*P-*value
RC continuous (mmol/l)	1718	1.60 (1.42 - 1.80)	< 0.001	1.60 (1.42 - 1.80)	< 0.001	1.14 (0.99 - 1.32)	0.072
RC quartile							
Q1 (< 0.46 mmol/)	430	Reference		Reference		Reference	
Q2 (0.47 - 0.88 mmol/)	428	1.52 (1.03 - 2.25)	0.036	1.50 (1.01 - 2.23)	0.043	1.39 (0.89 - 2.15)	0.147
Q3 (0.89 - 1.54 mmol/)	431	2.02 (1.39 - 2.95)	< 0.001	1.98 (1.35 - 2.89)	< 0.001	1.29 (0.84 - 1.99)	0.245
Q4 (1.55 - 5.32 mmol/)	429	3.69 (2.57 - 5.29)	< 0.001	3.64 (2.54 - 5.22)	< 0.001	1.73 (1.13 - 2.65)	0.012
*P* for trend			< 0.001		< 0.001		0.022

RC, remnant cholesterol; CI, confidence interval.

Unadjusted Model adjusted for none.

Model I adjusted for age, gender.

Model II adjusted for age, gender, duration of illness, comorbid anxiety, psychotic symptoms, HAMA, HAMD, TGAb, TSH, TPOAb, FBG, BMI, SBP and DBP.

### 3.3 Non-linear relationship analysis

**Figure 1:**
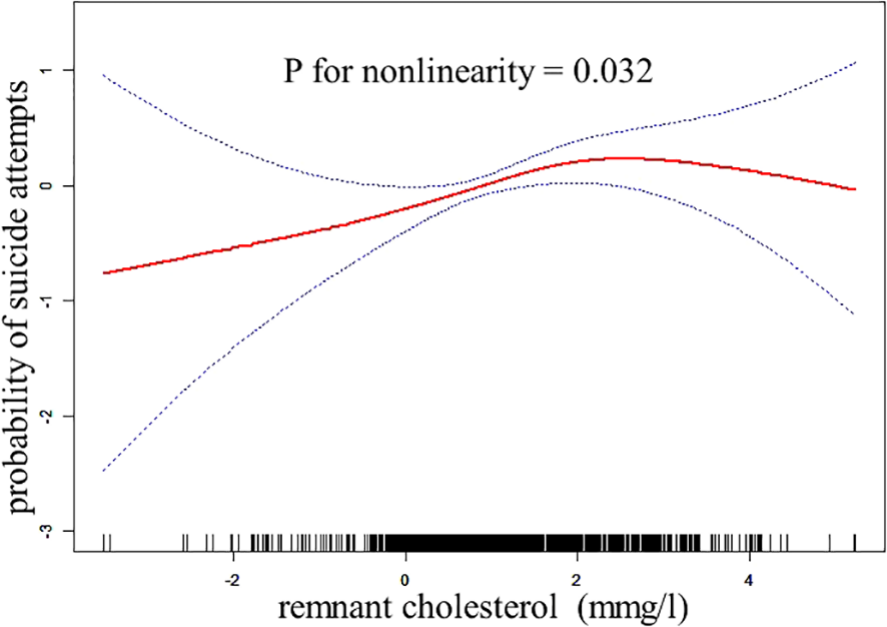
The relationship between RC and the probability of suicide attempts.

The findings of a two-piecewise logistic regression model examining the relationship between RC and SA are shown in **Table 3**. The inflection point of RC is 1.99 mmol/L. For participants with RC levels below 1.99 mmol/L, the odds ratio (OR) was 1.36 (95% CI: 1.09 to 1.69, p = 0.006), indicating a significant association between lower RC levels and an increased likelihood of SA. Conversely, for participants with RC levels equal to or greater than 1.99 mmol/L, the OR was 0.79 (95% CI: 0.55 to 1.14, p = 0.214), suggesting no significant association in this group. The difference in slopes between the two segments was calculated as 0.59 (95% CI: 0.36 to 0.96, p = 0.034), indicating a statistically significant change in the relationship between RC and SA at the inflection point. The predicted value at the inflection point of 1.99 mmol/L was -0.88 (95% CI: -1.12 to -0.64). The log-likelihood ratio test yielded a p-value of 0.032 (**Figure 1**), supporting the significance of the model. This analysis was adjusted for age, gender, duration of illness, comorbid anxiety, psychotic symptoms, HAMA, HAMD, TSH, TGAb, TPOAb, FBG, BMI, SBP, and DBP.

**Table 3:** The results of the two-piecewise logistic regression model.

Inflection points of RC	OR	95%CI	*P*-value
Inflection point	1.99		
< 1.99 slope 1	1.36	1.09 to 1.69	0.006
≥ 1.99 slope 2	0.79	0.55 to 1.14	0.214
slope 2 – slope1	0.59	0.36 to 0.96	0.034
predicted at 1.99	-0.88	-1.12 to -0.64	
Log likelihood ratio test			0.032

Effect: remnant cholesterol (RC); cause: suicide attempts (SA); adjusted for age, gender, duration of illness, comorbid anxiety, psychotic symptoms, HAMA, HAMD, TSH, TGAb, TPOAb, FBG, BMI, SBP and DBP.

### 3.4 Subgroup analysis between RC and SA

To elucidate the relationship between SA risk and different ages, subgroup analysis was conducted by stratifying patients based on age (18-29 years old, 30-39 years old, 40-49 years old, 50-60 years old) (**Table 4**). In the age subgroup, RC was significantly associated with SA risk only in the 19-29 age group (OR=1.29, 95% CI 1.01-1.64, P=0.040).

**Table 4:** Subgroup analysis between RC and SA.

	N	OR	95%CI	*P-*value
Age (years)	1718			
18-29	625	1.29	(1.01, 1.64)	0.040
30-39	404	1.40	(0.98, 2.00)	0.064
40-49	396	1.19	(0.87, 1.62)	0.269
50-60	266	0.78	(0.53, 1.16)	0.224

All adjusted for age at onset, duration of illness, education, comorbid anxiety, psychotic symptoms, HAMA, HAMD, TGAb, TSH, TPOAb, FBG, BMI, SBP and DBP.

## 4 Discussion

To the best of our knowledge, this is the first study to look at the relationship between SA and RC levels in a large population of Chinese patients who have major depressive disorder that is not being treated. The main findings of this study were as follows: (1) The incidence of suicide attempts was 20.1% (346/1718) in UFE MDD patients, with no statistically significant gender disparity observed. (2) We identified a novel non-linear relationship between RC levels and SA, characterized by a positive correlation below 1.99 mmol/L, with each unit increase in RC associated with a 36% higher SA risk. (3) Patients with the highest RC levels (Q4 group) demonstrated the longest disease duration, while age-stratified analysis revealed that RC-SA association was significant only in young adults (19-29 years).

There is a nonlinear correlation between suicide attempts and remnant cholesterol, according to our research. When RC < 1.99 mmol/L, each unit increase in RC was linked to a 36% increase in the risk factors of SA. The complex relationship between RC and SA likely involves multiple interconnected pathophysiological mechanisms: First, neuroinflammation plays a pivotal role in the pathophysiology of depression and suicidal behavior. Evidence suggests that elevated neutrophil-to-lymphocyte ratio (NLR) serves as a crucial factor in the etiology of MDD (40). Systematic reviews have identified NLR as a characteristic biomarker for suicide susceptibility among patients with MDD (41). Furthermore, postmortem studies of adolescent suicide victims have revealed increased levels of pro-inflammatory cytokines (IL-1β, IL-6, TNF-α) in the prefrontal cortex (42). RC may exacerbate this process by promoting inflammation and oxidative stress, leading to elevated pro-inflammatory cytokine levels, which are closely associated with suicidal ideation and attempts (43). Second, Insulin Resistance (IR) Mechanism. IR is closely linked to depression, with up to 50% of MDD patients exhibiting IR (44). A 9-year longitudinal study confirmed that increased IR indicators significantly correlate with higher MDD incidence (45). IR also impacts RC metabolism by impairing LRP1 transport to hepatocyte membranes, promoting RC accumulation, and increasing VLDL production via apoB degradation inhibition (17, 46). RC, associated with IR and metabolic dysfunction, may influence mood and suicidal behavior by disrupting brain glucose metabolism and energy homeostasis (47, 48). Furthermore, recent studies reveal a stable positive correlation between suicidal attempts (SA) and IR (triglyceride glucose index) in MDD patients, even after adjusting for confounding factors (49). Third, the Vascular Depression Hypothesis suggests that cerebral small vessel disease (CSVD), characterized by subcortical microvascular dysfunction, white matter hyperintensities, microbleeds, and lacunes in gray matter, predisposes to depression in the elderly through disruption of fronto-subcortical circuits (50). Meta-analytic evidence has substantiated this association, demonstrating that microvascular dysfunction contributes to late-life depression through compromised blood-brain barrier integrity and impaired cerebral perfusion (51, 52). Remnant cholesterol (RC) mediates this process by inducing endothelial dysfunction and reducing cerebral blood flow, disrupting mood-regulating circuits (53). Fourth, recent studies emphasize the crucial role of gut microbiota in mental disorders through the gut-brain axis (54, 55). The gut microbiota influences neurotransmitter production, particularly serotonin and dopamine (56), and systematic reviews have demonstrated associations between gut microbiota and various psychiatric disorders, including major depressive disorder, anxiety disorders, schizophrenia, and autism spectrum disorders (55). During states of anxiety, the gut microbiota can disrupt the tryptophan pathway and inhibit serotonin synthesis, which may contribute to the development and exacerbation of anxiety and depressive symptoms. Chronic stress can disturb gut microbiota homeostasis, creating a cycle that worsens anxiety symptoms and potentially increases suicide risk. Furthermore, gut microbiota influences cholesterol homeostasis through bile acid metabolism and short-chain fatty acid production, connecting cholesterol metabolism and mental health (57, 58).

The threshold effect observed in our study, where the association between RC levels and SA became non-significant above 1.99 mmol/L, warrants further discussion. This non-linear relationship suggests complex interactions between RC and suicidal behavior, potentially influenced by multiple factors. One possible explanation for this phenomenon is a saturation effect, where the impact of RC on neurobiological processes reaches a plateau after a certain concentration (59). This is consistent with recent findings in cardiovascular research, where the association between RC and atherosclerotic cardiovascular disease risk also exhibited a non-linear relationship (60). At higher RC levels, compensatory mechanisms may be activated to mitigate the adverse effects of elevated RC. For example, increased RC concentrations may trigger upregulation of anti-inflammatory pathways or enhance cholesterol efflux mechanisms, potentially attenuating the pro-inflammatory and neurotoxic effects of RC (61). These compensation mechanisms may involve the activation of liver X receptors (LXRs), which play a crucial role in cholesterol homeostasis and have been shown to have anti-inflammatory effects (62). Secondly, regarding the potential mechanisms, the comprehensive systematic review by Sen et al. (2022) provides crucial insights into the relationship between lipids and psychiatric conditions. Their findings particularly emphasize how low cholesterol levels may significantly increase the risk of violent suicide, which aligns with our observations. This analysis advanced our understanding of cholesterol-suicide mechanisms (63). Conversely, higher cholesterol levels may have a protective effect, helping to reduce suicide risk. High cholesterol levels can increase cell membrane viscosity, enhance synaptic plasticity, and improve serotonergic neurotransmission by promoting the release of 5-hydroxytryptamine (5-HT) (64), potentially reducing anxiety, depression, violence, and impulsive behavior, ultimately lowering suicide risk. Third, the effect of RC on suicide risk may be modulated by genetic variables. Numerous loci linked to RC levels have been found in recent genome-wide association studies, and these genetic differences may have an impact on the connection between RC and suicidal behavior (65). For example, changes in the genes APOA5 and LPL involved in triglyceride metabolism may affect the clearance of residual particles and their impact on neuroinflammation (66).

Our findings revealed no significant gender differences in suicide attempt prevalence among UFE patients with major depressive disorder, which contrasts with previous international studies reporting higher attempt rates in females (9) and higher completion rates in males (67). This discrepancy may be explained by China’s unique suicide patterns and their evolution. Epidemiological data from the 1990s demonstrated elevated suicide rates among females and rural residents in China, primarily attributed to limited rural medical resources, accessibility to lethal pesticides, and socio-cultural factors affecting women’s status (68). However, over the past two decades, this pattern has significantly shifted, with the male-to-female suicide ratio increasing due to declining female rates (69), suggesting diminishing gender influence in contemporary China’s suicide patterns. In addition, this study suggests that in intergroup comparisons of RC quartiles, patients in the Q4 group had the longest duration of untreated illness (7.1 ± 4.9 months) compared to other groups. Altamura et al. found that a longer duration of untreated illness (DUI) may negatively influence the clinical course of MDD (11). Recent research indicated that prolonged DUI was associated with elevated risks of anxiety, thyroid disorders, overweight, and hyperlipidemia in MDD patients (70). Prolonged duration of untreated illness (DUI) impacts patients by altering lifestyle, including disturbed appetite, reduced physical activity, and impaired sleep quality, which in turn disrupt lipid metabolism-related hormone secretion (71).

Furthermore, our age-stratified analysis revealed a positive correlation between RC and SA in the youngest group (18–29 years), consistent with prior findings. Xiao et al. (10) showed that MDD patients with earlier onset (18–44 years) had higher rates of suicidal ideation compared to later-onset cases (60–85 years). Similarly, Herzog et al. (72) reported higher suicidal ideation frequencies in early-onset MDD patients. Additionally, Wedervang-Resell et al. (73) found elevated TC/HDL-c levels in first-episode drug-naive patients with early-onset psychosis, suggesting subclinical dyslipidemia as a feature of early-onset MDD. These findings highlight the need for early intervention to improve outcomes.

There are various limitations to our investigation. First, lipid levels were assessed at a single time point, limiting our ability to evaluate the dynamic relationship between RC levels and suicide attempts. Secondly, as a cross-sectional study, we were unable to establish a causal link between RC and suicidal behavior, and further research is needed to confirm our findings. Third, interviews and medical records, which are not structured assessment tools for suicidal behavior, were used to identify suicide attempts. In addition, we were not able to provide precise information about the severity, modality, real threat, plans, thoughts, and timeframe of suicide attempts. It is very likely that such notions may be important in the discussion of suicidal risk. Therefore, in future studies, a specific suicide questionnaire should be used to collect information related to suicide attempts. Fourth, our study was the lack of family history data, including both psychiatric disorders (particularly suicide-related) and metabolic abnormalities. Given the well-documented familial aggregation of psychiatric disorders and suicidal behaviors, as well as the strong genetic predisposition to metabolic abnormalities, the absence of this information may have limited our ability to fully interpret the observed associations and control for potential confounding factors. Future research should include family history assessments to better account for these potential hereditary influences. Fifth, because our participants were Chinese, the findings may not be generalizable to other ethnic groups. Multicenter, multi-ethnic studies are needed to validate and extend these results. Sixth, our participants were adults aged 18–60 years, but age significantly influences cholesterol levels, potentially introducing bias. Larger studies across broader age ranges are necessary to confirm our findings. Finally, while our focus on untreated first-episode (UFE) patients offers unique insights, it also presents challenges. Depression may initially manifest in patients with unipolar depression or bipolar disorder. Although we conducted follow-up diagnoses at 3-6 months and only included patients diagnosed with MDD at both time points, we cannot definitively rule out the possibility of some diagnoses evolving into bipolar disorder. To address this limitation in future research, we propose extending the follow-up period or employing prospective diagnostic tools to enhance the identification of potential bipolar disorder.

The study concludes that among Chinese patients with UFE MDD, RC and SA have a nonlinear connection with an inflection point around 1.99 mmol/L. When RC < 1.99 mmol/L, there is a positive correlation between RC and SA at the left inflection point, but this correlation is not statistically significant on the right side. This finding highlights the complex interactions between lipid metabolism and suicidal behavior in depressed patients. While lower RC levels showed an association with increased suicide risk in UFE MDD patients, this parameter should be considered only as one of multiple risk indicators within a comprehensive suicide risk assessment framework. The relationship between RC levels and suicide risk requires further investigation, considering the complex nature of suicidal behavior and the multiple factors influencing lipid metabolism.

## Data Availability

The original contributions presented in the study are included in the article/supplementary material. Further inquiries can be directed to the corresponding authors.
